# Molecular Pathophysiology of Hepatocellular Carcinoma: From Metabolic Inflammation to Therapeutic Targets

**DOI:** 10.3390/cancers18142335

**Published:** 2026-07-20

**Authors:** Shady Azzam, Alexandra Straus, Can Senkal, Devanand Sarkar

**Affiliations:** Department of Cellular, Molecular and Genetic Medicine, Virginia Commonwealth University, Richmond, VA 23298, USA; azzamse@vcu.edu (S.A.); alexandra.straus@vcuhealth.org (A.S.); can.senkal@vcuhealth.org (C.S.)

**Keywords:** AEG-1, SND1, hepatocellular carcinoma (HCC), lipid metabolism, inflammation, MASH, MASLD, tumor microenvironment, nanomedicine, target discovery

## Abstract

**Background/Objectives:** Hepatocellular carcinoma (HCC) is undergoing a profound epidemiological shift from viral etiologies toward metabolic dysfunction-associated steatohepatitis (MASH). Current targeted therapies often fail to provide effective responses due to the complex, interconnected nature of the tumor microenvironment. This review aims to explain the molecular axis linking chronic metabolic injury to carcinogenesis, focusing specifically on the oncoproteins Astrocyte elevated gene-1/metadherin (AEG-1/MTDH) and staphylococcal nuclease and tudor domain-containing 1 (SND1) as cooperating regulators of this disease network. **Methods:** This article represents a narrative review of the published literature and does not follow a systematic or exhaustive search protocol. A structured literature search using specific search terms was conducted across PubMed, Scopus, and Web of Science databases, with a last search date of May 2026. **Results:** Available preclinical data indicate that AEG-1 mediates early preneoplastic injury via dysregulation of hepatic lipid metabolism leading to lipotoxicity and survival of genetically unstable hepatocytes. As the disease progresses, AEG-1 amplifies NF-κB-driven inflammation and, as tumors emerge, recruits SND1 as a cooperating partner to form a gene-silencing complex that suppresses tumor suppressor proteins. Furthermore, AEG-1 and SND1 reprogram surrounding macrophages into an immunosuppressive state and drive tumor resistance to standard anti-angiogenic and chemotherapeutic drugs. **Conclusions:** The evidence reviewed supports a model in which HCC is driven by an interconnected metabolic-inflammatory-oncogenic cycle. AEG-1 functions as an upstream metabolic and inflammatory driver that cooperates with SND1 in a subset of oncogenic silencing events within this pathogenic network. Therefore, utilizing advanced nanomedicine platforms to simultaneously target these proteins represents a mechanistically rational therapeutic strategy that warrants further preclinical evaluation in advanced HCC.

## Simple Summary

Metabolic conditions, such as fatty liver disease, are becoming the leading risk factor for primary liver cancer or hepatocellular carcinoma (HCC). Because HCC pathophysiology involves multifactorial processes of fat buildup, chronic inflammation, and tissue scarring, standard drugs that target only one problem usually fail to provide long-term treatment. In this review, we explain how two specific proteins, AEG-1 and SND1, act as cooperating regulators that connect all these processes together. We aim to show how these proteins help HCC survive metabolic stress, grow rapidly, and hide from the body’s immune system. A better understanding of this network may support the design of targeted delivery vehicles (nanomedicines) aimed at these regulators, which could offer a new strategy for treating advanced liver cancer.

## 1. Introduction

### 1.1. Hepatocellular Carcinoma (HCC): A Shifting Landscape of Risk Factors

Liver cancer, specifically hepatocellular carcinoma (HCC) which is the most common primary liver malignancy, remains a global health challenge worldwide, and continues to grow in incidence [1]. In 2022, liver cancer was the sixth most common cancer worldwide, accounting for 865,269 new cases, and was the third leading cause of cancer-related deaths globally [2]. Notably, HCC is the fastest increasing cause of cancer-related deaths in the USA since the early 2000s, reflecting the need for new strategies to address the causes and progression of the disease [3,4].

HCC accounts for approximately 90% of liver cancer cases [5]. Over 90% of HCC cases occur in the setting of chronic liver diseases, with cirrhosis from any etiology as the strongest risk factor [6]. Viral etiologies, particularly hepatitis B virus (HBV)—historically the dominant driver accounting for approximately 50% of HCC cases globally—and hepatitis C virus (HCV), are now stabilizing or declining, especially in East Asia and sub-Saharan Africa, largely attributable to antiviral interventions and vaccination programs [7,8,9].

In contrast, metabolic dysfunction-associated steatotic liver disease (MASLD), and its progressive form metabolic dysfunction-associated steatohepatitis (MASH), are emerging as dominant drivers of HCC, particularly in the West and in high-income countries [7]. Chronic alcoholism and diet-induced obesity serve as predisposing events for MASLD. MASLD diagnostic criteria require hepatic steatosis (>5%) and features of metabolic dysregulation, and its prevalence globally is 38%, while its prevalence among obese/overweight patients is around 50% [8,9]. Further, between 1990 and 1994 and 2010–2014, the proportion of MASLD cases increased from 21% to 69%, highlighting a profound epidemiological shift from viral to metabolic HCC [9,10].

A particularly important and clinically underappreciated feature of MASLD-HCC is its frequent development in the absence of cirrhosis, meaning a large proportion of at-risk patients fall outside standard surveillance guidelines [11]. The molecular drivers linking this non-cirrhotic metabolic injury to malignant transformation—including dysregulated lipid metabolism, insulin resistance, oxidative stress, and immune reprogramming—remain incompletely understood, necessitating a deeper mechanistic interrogation of the hepatocarcinogenic process [9,12].

This evolving landscape has redefined HCC as a disease increasingly rooted in systemic metabolic dysfunction and has necessitated a re-examination of the molecular mechanisms linking metabolic injury to inflammation and oncogenic transformation—the central focus of this review.

### 1.2. The Clinical Challenge in HCC

Despite advances in surveillance and therapy, HCC remains associated with poor clinical outcomes, largely due to late-stage diagnosis and limited treatment efficacy [13]. Early detection of HCC is critical for effective intervention strategies such as surgical resection, liver transplantation, or local ablation, while diagnosis at later stages makes intervention strategies limited [13,14]. While the clinical guidelines from the American Association for the Study of Liver Diseases (AASLD), European Association for the Study of the Liver (EASL), and Korean Association for the Study of the Liver (KASL) recommend semiannual surveillance of HCC for all patients with cirrhosis, the few studies that have assessed the effectiveness of surveillance in patients with MASLD found no significant association between surveillance and the applicability of curative treatments [15,16,17]. This may be due to the lack of cirrhosis or liver disease diagnosis in a large proportion of MASLD-HCC patients, which would exclude them from the at-risk population targeted by surveillance guidelines [17,18]. Other studies have found that even when surveillance is performed using ultrasounds, inadequate visualization and subsequent surveillance failure were more likely in MASLD/MASH-HCC patients, leading to higher rates of late-stage HCC diagnosis [19,20].

Beyond challenges in early detection, therapeutic resistance remains a major barrier to effective HCC treatment. Tyrosine kinase inhibitors (TKIs) such as Sorafenib and Lenvatinib, have become standard treatment options; however, their effectiveness often extends survival by only a few months. Immune checkpoint inhibitors have provided new hope for patients with HCC [21]. The phase III IMbrave150 trial demonstrated that atezolizumab, an anti-programmed cell death ligand-1 (anti-PD-L1) antibody, in combination with bevacizumab, an anti-vascular endothelial growth factor-A (anti-VEGFA) antibody, improved survival for patients with advanced HCC compared to standard treatment with Sorafenib [22,23,24]. The phase III HIMALAYA trial subsequently established the STRIDE regimen—a single priming dose of tremelimumab [anti-cytotoxic T-lymphocyte–associated protein 4 (anti-CTLA-4) antibody] followed by regular interval durvalumab (anti-PD-L1 antibody)—as another first-line standard for unresectable HCC, with unprecedented 5-year overall survival data showing 20% of patients alive at five years compared to 9.4% in the Sorafenib arm [25,26,27]. Recently, the Food and Drug Administration (FDA) has approved nivolumab (anti-PD-1 antibody) and ipilimumab (anti-CTLA-4 antibody) combination for unresectable or metastatic HCC [28,29]. However, immune checkpoint inhibitors have shown limited responsiveness in patients, with only 15–20% of patients showing improved survival [13,30]. This limited response rate is at least partly attributable to the immunosuppressive tumor microenvironment characteristic of MASH-driven HCC, where metabolic inflammation, tumor-associated macrophage (TAM) polarization, and T cell exhaustion collectively impair anti-tumor immunity highlighting the need for therapeutic strategies that address the underlying metabolic-inflammatory drivers of immune evasion [30,31].

Sorafenib, the first multi-target kinase inhibitor for advanced HCC, extended overall survival in patients but was limited by the onset of resistance due to genetic mutations including Epidermal Growth Factor Receptor (EGFR) amplification and compensatory activation of phosphoinositide 3-kinase/Akt kinase (PI3K/AKT) and Wnt/β-catenin signaling pathways [32,33,34,35]. Regorafenib, a second-line multi-kinase inhibitor approved following sorafenib failure, demonstrated a modest but significant survival benefit in the RESORCE trial, with a systematic review and meta-analysis confirming its activity across real-world HCC populations [36,37]. Collectively, these molecular changes sustain tumor cell survival despite therapeutic pressures and enrich the cancer stem cell (CSC) population following long-term Sorafenib treatment, resulting in resistance, increased stemness, and enhanced tumor recurrence [35]. Lenvatinib, another anti-angiogenic multi-targeting TKI also approved for advanced HCC, similarly develop resistance, though studies suggest that co-treatment with immunotherapy may delay, but not mitigate, its onset [38,39,40]. Importantly, therapeutic response in MASH-HCC may differ from viral or alcohol-related HCC due to distinct immune phenotypes, metabolic comorbidities, and liver reserve constraints, highlighting the need for etiology-specific treatment selection strategies.

The combined challenges of late-stage diagnosis and therapeutic limitations underscore the need for deeper mechanistic understanding and innovative treatment strategies in HCC and MASLD/MASH-associated HCC. Such insights are essential for the development of effective screening and diagnosis strategies as well as targeted therapies, particularly in the expanding population of patients with metabolically driven liver disease.

### 1.3. Scope of the Review

This review aims to explain the molecular axis linking chronic metabolic injury to inflammation to carcinogenesis, with a particular focus on the upstream molecular driver Astrocyte elevated gene-1/Metadherin (AEG-1/MTDH) and its interacting partner Staphylococcal nuclease and tudor domain-containing 1 (SND1). This article represents a narrative review of the published literature and does not follow a systematic or exhaustive search protocol. A structured literature search was conducted across PubMed, Scopus, and Web of Science databases, with a last search date of May 2026. The following search terms were used individually and in combination: “hepatocellular carcinoma,” “HCC,” “AEG-1,” “MTDH,” “metadherin,” “SND1,” “staphylococcal nuclease domain containing 1,” “MASH,” “MASLD,” “NAFLD,” “NASH,” “metabolic dysfunction-associated steatohepatitis,” “lipotoxicity,” “endoplasmic reticulum stress,” “tumor microenvironment,” “fibrogenesis,” “immune evasion,” “angiogenesis,” “NF-κB,” “therapeutic targeting,” “nanomedicine,” “siRNA delivery,” “GalNAc,” and “lipid nanoparticle.” Articles were included if they reported original research, clinical trials, or prior reviews directly relevant to the molecular pathophysiology of MASH-driven HCC, or the biological and therapeutic roles of AEG-1 and SND1 in the setting of lipid metabolism, cellular stress, immune system activation, fibrogenesis, and therapeutic resistance. No language restrictions were applied; however, emphasis was placed on literature published within the last fifteen years to ensure contemporary relevance. As is inherent to narrative reviews, the selection and synthesis of references reflect the authors’ expert appraisal of the field, and the possibility of selection bias is acknowledged.

#### 1.3.1. Astrocyte Elevated Gene-1/Metadherin (AEG-1/MTDH)

AEG-1, also known as metadherin (MTDH), was originally identified as an HIV-1-inducible gene in primary human fetal astrocytes [41]. It was subsequently discovered to be massively overexpressed in a variety of solid tumors, including HCC, often driven by genomic amplification of chromosome 8q [42,43]. It is a downstream gene of Ha-ras, and transcriptionally regulated by c-Myc upon Ha-ras and PI3K activation [44]. Overexpressed AEG-1 functions as a bona fide oncogene promoting the hallmarks of cancer [45].

Structurally, AEG-1 is a single-pass transmembrane protein that localizes primarily to the endoplasmic reticulum (ER) membrane [42,46]. AEG-1 is not an enzyme and lacks a defined catalytic pocket. Instead, it functions as a versatile scaffold and adaptor protein. Because it operates almost entirely through protein–protein interactions (PPIs) as well as via protein-RNA interactions, AEG-1 acts as a scaffolding platform that brings together diverse signaling molecules to amplify stress responses, inflammation, and survival pathways [47,48].

#### 1.3.2. Staphylococcal Nuclease and Tudor Domain-Containing 1 (SND1)

SND1 is a highly conserved, multifunctional protein best known as a core component of the RNA-induced silencing complex (RISC) [49]. Unlike the scaffolding nature of AEG-1, SND1 has intrinsic endonuclease activity, which allows it to execute RNA interference (RNAi)-mediated gene silencing. Structurally, its enzymatic function is heavily reliant on tandem repeats known as SN1 and SN2 barrels. SND1 is frequently upregulated in HCC, where it contributes to the silencing of tumor suppressor mRNAs [50,51]. Additionally, SND1 has also been shown to participate in regulating mRNA splicing, editing and stability as well as miRNA processing [52,53,54,55,56]. In the nucleus, SND1 functions as a transcription coactivator for several transcription factors, such as signal transducer and activator of transcription 5 and 6 (STAT5 and STAT6), Epstein–Barr virus nuclear protein 2 (EBNA2) and c-Myb protooncogene (MYB) [57,58,59,60,61].

#### 1.3.3. The AEG-1/SND1 Complex

While both proteins independently promote tumorigenesis, their pathogenic synergy in HCC is driven by direct physical interaction [50]. AEG-1 physically binds to SND1 to form active RISC. By functioning as the scaffolding anchor for SND1’s endonuclease activity, AEG-1 directly enhances oncogenic gene silencing [50]. In addition, by interacting with SND1, AEG-1 increases SND1 protein stability, further augmenting SND1 oncogenic function [62].

Understanding this structural context, AEG-1 as an ER-resident stress sensor and scaffolding hub, and SND1 as its enzymatic partner, provides the necessary foundation for exploring how these proteins commandeer liver biology. As detailed in the following sections, this dysregulation begins at the earliest stages of chronic liver injury through the profound rewiring of hepatic lipid metabolism.

## 2. The Pre-Neoplastic Niche: Mechanisms of Injury

### 2.1. Metabolic Stress and Lipotoxicity

**Figure 1:**
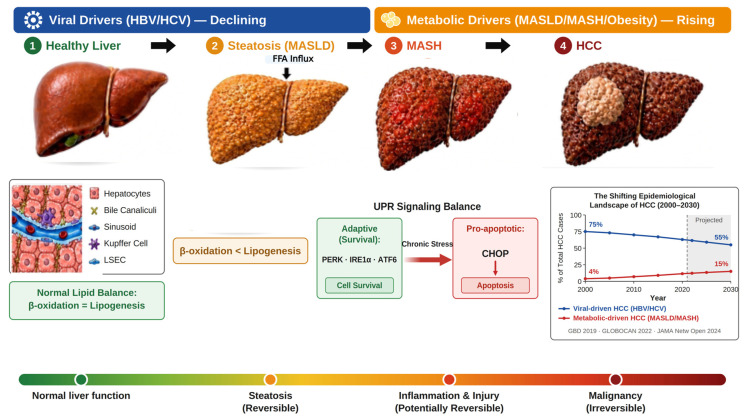
The pathogenic progression and shifting epidemiology of Hepatocellular Carcinoma (HCC). As viral drivers decline, metabolic dysfunction-associated steatotic liver disease (MASLD)/metabolic dysfunction-associated steatohepatitis (MASH) increasingly drives the transition to malignancy across four stages: (1) Healthy Liver: Normal lipid homeostasis (β-oxidation balances lipogenesis) and standard cellular architecture. (2) Steatosis (MASLD): Free fatty acid (FFA) influx disrupts metabolic balance, causing reversible lipid accumulation. (3) MASH: Chronic stress exhausts adaptive Unfolded Protein Response (UPR) survival mechanisms [PKR-like endoplasmic reticulum kinase (PERK), inositol-requiring enzyme 1 alpha (IRE1α), activating transcription factor 6 (ATF6)], shifting the liver toward C/EBP homologous protein (CHOP)-mediated apoptosis and inflammation. (4) HCC: Irreversible malignancy, highlighting a distinct “Non-Cirrhotic Pathway” where metabolic HCC develops directly from MASH without preceding cirrhosis. Schematic prepared by the authors. Visualization was ceated by BioRender (https://www.biorender.com/ accessed on 25 May 2006).

The pathogenesis of MASH is multifactorial but primarily involves chronic disruption of hepatic lipid metabolism and homeostasis (Figure 1) [63]. Under physiological conditions, the liver maintains lipid balance through coordinated acquisition of lipids via free fatty acid (FFA) uptake and de novo lipogenesis, which is offset by lipid elimination via mitochondrial β-oxidation and very low-density lipoprotein (VLDL) export. However, in the context of metabolic syndrome, peripheral insulin resistance impairs insulin’s ability to suppress lipolysis, resulting in increased breakdown of triglycerides into FFAs and glycerol, causing a massive influx of circulating FFAs into hepatocytes [64].

Initially, the liver attempts to manage this excess by esterifying FFAs into inactive triglycerides stored within lipid droplets, the hallmark feature of steatosis. However, continuous influx eventually overwhelms this buffering capacity, leading to the accumulation of lipotoxic intermediates—including ceramides, diacylglycerols (DAGs), and lysophosphatidylcholines (LPCs) [65]. These toxic lipid species compromise mitochondrial integrity, uncoupling the electron transport chain, impairing oxidative phosphorylation and ATP synthesis, and driving dysregulated generation of reactive oxygen species (ROS) that perpetuate oxidative stress. This lipotoxicity is the primary pathophysiological catalyst that transitions benign steatosis into the necro-inflammatory state of MASH [63,66].

**Figure 2:**
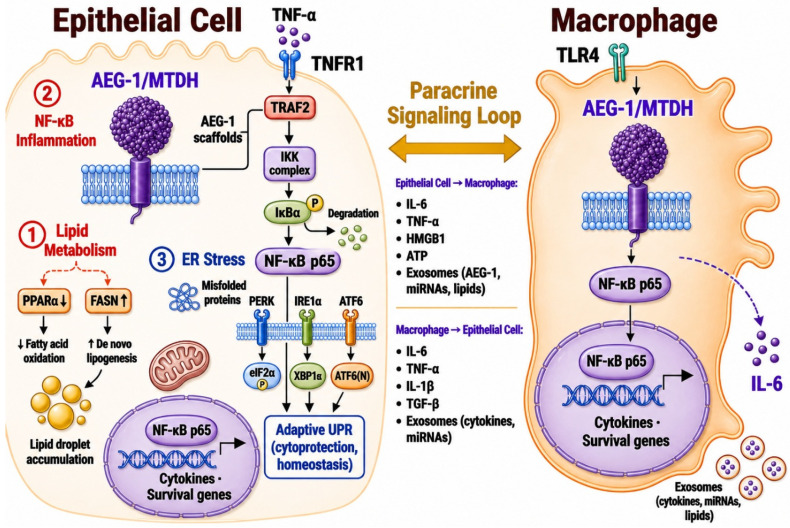
Structural localization and multi-pathway signaling of Astrocyte elevated gene-1/metadherin (AEG-1/MTDH) in epithelial-macrophage crosstalk. AEG-1 acts as a regulatory node for intracellular stress and intercellular communication. (**Left**) Epithelial Cell: As a type II endoplasmic reticulum [ER] membrane protein, AEG-1 modulates three primary cascades: (1) Lipid metabolism: Promotes lipid droplet accumulation by suppressing peroxisome Proliferator-Activated Receptor alpha (PPAR-α) and upregulating fatty acid synthase (FASN). (2) Nuclear factor kappa B (NF-κB) and inflammation: Enhances tumor necrosis factor receptor 1 (TNFR1)-mediated activation of the TNF receptor-associated factor 2 (TRAF2) and IκB kinase (IKK) complex, allowing p65 subunit of NF-κB to translocate to the nucleus and NF-κB to transcribe inflammatory and survival genes. (3) ER Stress: Misfolded proteins trigger the Unfolded Protein Response via three sensors [PKR-like endoplasmic reticulum kinase (PERK), inositol-requiring enzyme 1 alpha (IRE1α), activating transcription factor 6 (ATF6)] for adaptive cytoprotection. (**Center**) Paracrine loop: A bidirectional exchange sustains the inflammatory microenvironment. Epithelial cells release cytokines, damage-associated molecular patterns (DAMPs), and exosomes to activate macrophages, which reciprocate with potent pro-inflammatory and fibrogenic signals [interleukin 6 (IL-6), tumor necrosis factor alpha (TNF-α), interleukin 1 beta (IL-1β), transforming growth factor beta (TGF-β)]. (**Right**) Macrophage: Toll-like receptor 4 (TLR4) engagement and intrinsic AEG-1 activity amplify NF-κB transcription, driving robust IL-6 secretion to perpetuate the paracrine injury cycle. Schematic prepared by the authors. Visualization was created with the assistance of Perplexity.ai (https://www.perplexity.ai/, accessed on 25 May 2006).

While this biochemical sequence of lipotoxic injury is well established, the underlying genetic and molecular triggers that initiate and sustain this metabolic collapse are now being revealed. One candidate bridging this gap is AEG-1. AEG-1 has been primarily studied as an oncogene driving the progression of HCC [43,67,68,69]. Independent clinicopathological analysis of a tissue microarray from 323 HCC patients confirmed that AEG-1 overexpression correlates with microvascular invasion, poor differentiation and reduced overall survival [70]. More recent work has implicated AEG-1 not only as a cancer promoter but also as an early-stage metabolic disruptor (Figure 2) [71]. In vivo studies provide strong evidence: transgenic mice engineered to overexpress AEG-1 specifically in hepatocytes (Alb/AEG-1) develop spontaneous MASH, while AEG-1 conditional knockout mice are protected from high-fat diet-induced steatohepatitis [71]. AEG-1 actively drives this lipotoxic phenotype through a bidirectional dysregulation of lipid metabolism. Via its LXXLL nuclear receptor-binding motif, AEG-1 physically interacts with and inhibits Peroxisome Proliferator-Activated Receptor alpha (PPARα), a principal transcriptional regulator of hepatic lipid catabolism, impairing the hepatocyte’s capacity for mitochondrial β-oxidation and lipid clearance [71,72,73]. However, this motif does not act as a simple pro-disease element: in an AEG-1-L24K/L25H knock-in mouse in which the LXXLL motif is mutated to LXXKH, PPARα activation is restored and animals are partially protected from high-fat diet-induced steatosis, yet the same livers display increased lipogenic enzyme expression, mitogenic activity, and inflammation [74]. This dissociation, partial protection from steatosis offset by increased lipogenesis, mitosis and inflammation, will be discussed further in Section 5.2 to explain why targeting the LXXLL/PPARα interface is an unsuitable therapeutic strategy. Concomitantly, AEG-1 promotes the preferential association of Fatty Acid Synthase (FASN) mRNA with polysomes, thereby enhancing its translation and driving de novo lipogenesis even under conditions of existing lipid excess [71,74]. By simultaneously accelerating lipid synthesis and impairing lipid clearance, these findings position AEG-1 as a proximal driver of the metabolic stress and ROS generation that precipitates advanced liver injury.

Studies have also unraveled the role of SND1 in regulating lipid metabolism. SND1 was detected in lipid droplets in multiple species, suggesting a potential role in regulating lipid metabolism [75]. Overexpression of SND1 in rat primary hepatocytes and HCC cells showed increased phospholipids and cholesterol esters (CE) [76,77,78]. SND1 overexpression increased the activity of sterol regulatory element binding transcription factor 2 (SREBF2/SREBP2), which is a major regulator of cholesterol metabolism, while SREBP2 itself binds to SND1 promoter positively regulating its transcription, demonstrating a feed-forward loop [77,79]. However, SND1-overexpressing global transgenic mice did not show increased hepatic steatosis upon high-fat diet feeding even though gene expression analysis showed increased SREBP2 activity in the liver [80]. It was suggested that in the global transgenic mice, SND1 overexpression preserved pancreas function, so that these mice were insulin-sensitive which protected them from steatosis, while the WT littermates were insulin-resistant and developed steatosis [80]. We generated hepatocyte-specific SND1 transgenic mice (Alb/SND1) which showed features of mild steatosis at one year of age, compared to the WT littermates [51]. However, the magnitude of steatosis development in Alb/SND1 mice were much less than that observed in Alb/AEG-1 mice [51,71]. Whole body SND1 knockout mouse did not show any noticeable phenotype [81]. Additionally, high-fat diet feeding induced similar phenotypes in conditional hepatocyte-specific SND1 knockout mice and their WT littermates [82]. These findings suggest that unlike AEG-1, SND1 may not regulate basal lipid metabolism, and it participates in this process only when overexpressed. The underlying molecular mechanism by which SND1 regulates lipid metabolism remains to be deciphered. Does SND1 physically interact with SREBP2 and function as a transcription coactivator as it does for other transcription factors? Is enzymatic activity of SND1 required for regulating SREBP2 function? These questions need to be addressed in future studies.

### 2.2. Cellular Stress Responses: Endoplasmic Reticulum (ER) Stress and the Unfolded Protein Response

The lipotoxicity and oxidative stress that define MASH compromise the protein-folding capacity of the endoplasmic reticulum (ER). Hepatocytes are vulnerable to ER stress given their role as the primary site of lipid and protein synthesis [83]. Persistent accumulation of toxic lipid intermediates and ROS disrupts ER calcium homeostasis and redox balance, resulting in the accumulation of misfolded proteins within the ER lumen—a state that triggers the Unfolded Protein Response (UPR) [84].

The UPR initially mounts an adaptive response to restore ER proteostasis. Dissociation of the chaperone heat shock protein family A (Hsp70) member 5 (HSPA5/BiP/GRP78) from three ER transmembrane sensors, endoplasmic reticulum to nucleus signaling 1/Inositol-Requiring Enzyme 1α (ERN1/IRE1α), eukaryotic translation initiation factor 2 alpha kinase 3/Protein Kinase RNA-like ER Kinase (EIF2AK3/PERK), and Activating Transcription Factor 6 (ATF6), initiates three parallel signaling branches. ATF6 undergoes Golgi-dependent proteolytic cleavage to release a transcriptional activator that upregulates ER chaperones, including BiP/GRP78 and heat shock protein 90 beta family member 1 (HSP90B1/GRP94), expanding folding capacity [85]. Concurrently, PERK-mediated phosphorylation of eukaryotic translation initiation factor 2 subunit alpha (EIF2S1/eIF2α) attenuates global mRNA translation to reduce the incoming protein load, while IRE1α mediates unconventional splicing of X-box binding protein 1 (XPB1) mRNA, generating the active transcription factor XBP1s, which upregulates ER-associated degradation (ERAD) components to clear misfolded proteins [85].

In the setting of chronic metabolic injury, this adaptive response becomes exhausted [86]. Prolonged ER stress shifts UPR signaling from a pro-survival to a pro-apoptotic program. This transition is driven by sustained mitogen-activated protein kinase 8 (MAPK8/JNK) activation downstream of IRE1α and upregulation of the transcription factor DNA damage inducible transcript 3/C/EBP Homologous Protein (DDIT3/CHOP). CHOP suppresses anti-apoptotic protein B cell leukemia/lymphoma 2 (BCL2) while inducing the pro-apoptotic effectors BCL2 like 11 (BCL2L11/BIM) and BCL2 binding component 3 (BBC3/PUMA), tipping the mitochondrial apoptotic balance toward cell death and committing damaged hepatocytes to apoptosis [86].

For MASH to progress to HCC, a subset of hepatocytes must acquire the capacity to evade CHOP-mediated apoptosis despite chronic lipotoxic exposure. AEG-1 has been identified as an oncogenic regulator of this dysregulated survival [48,87]. AEG-1 is a transmembrane protein that localizes to the ER membrane and is induced by the cellular stresses that activate the UPR, positioning it within these stress-response pathways [46]. Hepatocytes overexpressing AEG-1 show resistance to stress-induced cell death, decoupling chronic ER stress from its apoptotic outcome [67]. Conversely, deletion of AEG-1 makes them more susceptible to stress [88]. This pro-survival effect operates through AEG-1-driven activation of the PI3K/AKT axis, with corresponding increases in anti-apoptotic proteins BCL2, myeloid cell leukemia sequence 1 (MCL1), X-linked inhibitor of apoptosis (XIAP) and baculoviral IAP repeat containing 5 (BIRC5/Survivin) and decreases in pro-apoptotic mediators BCL2-associated agonist of cell death (BAD) and cyclin-dependent kinase inhibitor 1A (CDKN1A/p21) [67,68]. AEG-1 also interacts with mouse double minute 2 homolog (MDM2) via its N-terminal domain, preventing MDM2 self-ubiquitination and stabilizing the protein, thereby sustaining MDM2-mediated p53 proteasomal degradation and suppressing the p53 apoptotic program [89,90]. As an ER-resident RNA-binding protein whose interactome is enriched in ER-resident protein-encoding mRNAs, AEG-1 further implicates itself in modulating ER proteostatic machinery to sustain cellular viability [46]. A recent study documented direct interaction between AEG-1 and IRE1α, although more in-depth functional studies are required to unravel the significance of this interaction, especially in the context of hepatocytes [91]. Through these mechanisms, AEG-1 may enable genetically unstable hepatocytes to persist and proliferate, creating a permissive context for malignant transformation.

### 2.3. Modes of Cell Death

The lipotoxicity and unresolved ER stress ultimately force the damaged hepatocyte toward cell death. However, in the context of MASH, cell death is not a quiet removal of a defective cell, it is the primary pathogenic cascade that drives chronic liver inflammation [63]. The specific way a hepatocyte dies dictates the magnitude of the surrounding immune response, creating a microenvironment that actively selects precancerous clones.

Historically, apoptosis was considered the primary mode of cell death in chronic liver disease [86]. Driven by caspase activation, apoptosis is generally a controlled, immunologically silent process in which the cell is systematically dismantled, with its contents packaged into apoptotic bodies for efficient efferocytosis. In the lipotoxic environment of MASH, however, this disposal system is overwhelmed. Recent evidence highlights the dominance of inflammatory, lytic forms of programmed cell death, specifically necroptosis and ferroptosis. Necroptosis ruptures the cell membrane via the receptor interacting serine/threonine kinase 1/receptor interacting serine/threonine kinase 3/mixed lineage kinase domain like pseudokinase (RIPK1/RIPK3/MLKL) pathway, spilling immunostimulatory damage-associated molecular patterns (DAMPs) into the hepatic parenchyma, activating the innate immune system [92]. Similarly, because MASH is defined by a surplus of toxic lipids, hepatocytes are vulnerable to ferroptosis, an iron-dependent death driven by lipid peroxidation [93]. Ferroptosis is executed through the failure of glutathione peroxidase 4 (GPX4), the sole enzyme capable of reducing cytotoxic phospholipid hydroperoxides when its obligate substrate, reduced glutathione (GSH), is depleted by chronic oxidative stress [94]. GSH availability is further constrained by suppressed cystine import via the system Xc^−^ transporter solute carrier family 7 member 11 (SLC7A11). The MASH liver amplifies this vulnerability through iron dysregulation generating Fenton-reactive Fe^2+^, enrichment of membrane phospholipids with polyunsaturated fatty acid chains susceptible to lipoxygenase-driven peroxidation, and suppression of the ferroptosis suppressor protein 1 (FSP1)/ubiquinol defense axis [94]. The resulting release of oxidized lipid DAMPs activates NLR family pyrin domain containing 3 (NLRP3) inflammasome assembly and Kupffer cell pro-fibrogenic signaling, propagating the sterile inflammatory cascade that sustains the pre-neoplastic niche. Together, these lytic death pathways sustain a cycle of injury, cytokine release, and compensatory regeneration.

This cycle of cellular destruction and forced cell division creates a mutagenic milieu, establishing a microenvironment permissive to the clonal expansion of genomically unstable hepatocytes. Yet, for a mutated hepatocyte to progress into hepatocellular carcinoma, it must overcome a fundamental selective barrier, acquiring resistance to the lipotoxic cell death eliminating adjacent hepatocytes.

Among the molecular regulators capable of conferring this resistance, AEG-1 has emerged as a mediator of dysregulated hepatocyte survival. When upregulated by the stressed microenvironment, AEG-1 activates cellular survival cascades, especially PI3K/AKT and Nuclear factor kappa-light-chain-enhancer of activated B cells (NF-κB) [43,67,68,95,96,97,98,99]. Through these networks, AEG-1 upregulates anti-apoptotic effectors while neutralizing death-inducing signals [67]. By uncoupling the precancerous hepatocytes from the microenvironmental pressures that eliminate adjacent cells, AEG-1 enables genomically unstable clones to persist and proliferate, driving the transition from chronic injury to malignancy [48]. The relationship between AEG-1 and the lytic cell death modalities predominant in MASH (necroptosis and ferroptosis) remains incompletely characterized. AEG-1’s scaffolding of K63-ubiquitinated RIPK1 within the NF-κB activation complex [85,86,87] raises the mechanistically plausible but untested hypothesis that AEG-1 may modulate the threshold between NF-κB pro-survival signaling and RIPK3/MLKL-dependent necrosome assembly downstream of TNFR1 engagement; however, no published study has directly examined AEG-1-mediated regulation of RIPK3 activation or MLKL phosphorylation in hepatocytes. Similarly, a direct causal role for AEG-1 in ferroptosis suppression, potentially through PI3K/AKT-driven NRF2/SLC7A11/GPX4 induction has not been established in MASH-relevant models. Resolving AEG-1’s functional position within these death-resistance circuits represents a high-priority mechanistic question for future investigation in etiology-specific in vivo systems. AEG-1 also functions as an inhibitor of senescence, which functions as a barrier to early HCC by inhibition proliferation of damaged or precancerous hepatocytes, thereby facilitating expansion of these cells [67,68].

## 3. Tumor Microenvironment and HCC

The transition from chronic metabolic injury to HCC is not only a consequence of accumulated genomic damage within hepatocytes, but it is also actively facilitated by a pro-oncogenic inflammatory microenvironment that develops with cellular transformation [100,101]. The liver’s resident immune cells (such as Kupffer cells), matrix-producing hepatic stellate cells, and intercellular networks that link these compartments together establish the tumor microenvironment (TME) [102]. TME enables and sustains HCC. Within this TME, the oncoproteins AEG-1 and SND1 have been implicated not only in hepatocyte transformation, but also in immune activation, fibrogenesis, and immunosuppression.

### 3.1. Innate Immune Activation

Kupffer cells (KCs) are liver resident macrophages which function as the primary guards of the hepatic parenchyma and represent about 20% of all hepatic cells [103]. Under physiological conditions, KCs maintain hepatic homeostasis by clearing debris, pathogens, and apoptotic material. However, during chronic metabolic injury such as MASH, KCs are constantly exposed to DAMPs such as oxidized lipids, high mobility group box 1 (HMGB1) and mitochondrial DNA released from injured hepatocytes [92]. Pattern recognition receptor engagement, via toll-like receptor 4 (TLR4), triggers NF-κB-dependent transcription of pro-inflammatory cytokines including interleukin-6 (IL6), tumor necrosis factor alpha (TNF-α), and interleukin-1 b (IL-1β) [101,104]. This cytokine storm acts on adjacent hepatocytes to activate oncogenic STAT3 signaling, promoting the proliferation and survival of the transformed cells [105,106]. This completes a pathogenic amplification loop that links sterile innate immune activation to development of hepatocellular cancer [106].

In the nucleus, AEG-1 acts as a bridging factor between the p65 subunit of NF-κB and the coactivator CREB-binding protein (CBP), directly amplifying NF-κB-driven transcriptional output [95,96]. ER membrane-anchored AEG-1 associates with lysine-63 (K63)-ubiquitinated signaling intermediates, such as receptor interacting serine/threonine kinase 1 (RIPK1) and TNF receptor associated factor 2 (TRAF2), stabilizing their accumulation and sustaining IkB kinase (IKK) complex activation [97]. AEG-1 is itself a direct substrate of IKKβ, the same kinase responsible for IκBα degradation [99]. These interactions establish AEG-1 as an integral component of the primary NF-κB activation complex rather than a secondary regulatory factor. This multilevel engagement of the NF-κB cascade is particularly important by the observation that AEG-1 mRNA is expressed approximately four-fold higher in KCs than in hepatocytes in the naive adult mouse liver, suggesting that AEG-1’s inflammatory role in the macrophage may be even more dominant than its well-known hepatocellular oncogenic role [88]. Consistent with this, macrophage-intrinsic AEG-1 has been reported to be a major contributor to hepatic IL-6 production and HCC promotion [88]. In experimental models, myeloid-specific AEG-1 deficiency (AEG-1^Δ^^MAC^ mice) results in near-complete protection from hepatocarcinogenesis and a marked suppression of hepatic IL-6 levels than hepatocyte-specific deficiency [88]. More importantly, myeloid-specific AEG-1 deletion also protects mice from development of MASH, supporting a role for AEG-1-driven inflammation across the disease spectrum, from MASH to HCC [107]. Inflammation in mesenteric fat was significantly decreased in high-fat, high-sugar-fed AEG-1^ΔMAC^ mice compared to AEG-1^fl/fl^ mice [107]. While conditioned medium (CM) from AEG-1^fl/fl^ KC activated stellate cells and induced pro-inflammatory cytokines in adipocytes, CM from AEG-1^ΔMAC^ KC failed to do so [107]. The immune-generated IL-6 targets STAT3 in neighboring hepatocytes to upregulate further AEG-1 expression, creating a vicious positive feedback loop [71,88,107]. Together, these findings support a role for AEG-1 not only as a hepatocellular oncogene but also as an inflammatory mediator operating simultaneously across both the parenchymal and immune compartments of the liver. The pro-inflammatory role of AEG-1 has been shown to be a driving factor in additional cancers, such as gastric cancer, and pre-neoplastic lesions, such as colitis [108,109].

### 3.2. Fibrogenesis and Hepatic Stellate Cell Activation

In parallel with the macrophage-driven cytokine storm, chronic hepatic injury activates a fibrogenic program carried out by hepatic stellate cells (HSCs), the liver’s principal scar-forming cells. In a healthy liver, HSCs reside quietly in the space of Disse, storing vitamin A and helping regulate blood flow through the sinusoids [110]. However, when the liver is under sustained injury as in MASH, HSCs receive distress signals, such as transforming growth factor β1 (TGF-β1), platelet-derived growth factor-BB (PDGF-BB), TNF-α, and IL-1β, from surrounding damaged hepatocytes and activated macrophages that cause them to leave their resting state and transform into activated, scar-producing cells [110,111]. This transformation is marked by loss of lipid droplets, development of contractile properties through expression of α-smooth muscle actin (α-SMA), and upregulation of collagen synthesis. While TGF-β1 is the primary signal driving HSC activation and collagen synthesis, PDGF-BB, secreted by activated KCs and injured hepatocytes, serves as the dominant mitogenic signal, expanding the population of scar-forming HSCs in proportion to the severity of liver injury [110].

At the molecular level, TGF-β1 induces HSC activation through binding to the TGFBR1/TGFBR2 receptor complex resulting in phosphorylation of the intracellular mediators SMAD family member 2 (SMAD2) and SMAD3 [112]. Phosphorylated SMAD3 partners with SMAD4 and moves into the nucleus, where it turns on genes encoding ECM proteins (collagen type I and III). There is also upregulation of tissue inhibitor of metalloproteinases (TIMPs), which block the enzymes that would normally break down the accumulating collagen [112]. The result is a self-amplifying fibrogenic circuit in which more collagen is made and less is degraded. In MASH specifically, injured hepatocytes compound this effect by simultaneously releasing Sonic Hedgehog (SHH) ligands that potentiate SMAD3 activity in HSCs, highlighting the synergistic effect of multiple hepatocyte-derived pro-fibrogenic signals beyond TGF-β1 alone [111]. Importantly, AEG-1 overexpression in hepatocytes has been shown to induce TGF-β1 expression and SMAD2 and SMAD3 phosphorylation providing a direct mechanistic link between AEG-1-driven metabolic injury and the paracrine fibrogenic signal delivered to HSCs [68,71]. TGF-β1 or lipopolysaccharide (LPS) treatment induces AEG-1 expression in HSCs [113]. Knockdown of AEG-1 in HSCS downregulate PI3K/Akt signaling dampening their proliferation and induction of apoptosis with simultaneous inhibition of collagen I and α-SMA expression [113]. Conditioned medium from AEG-1-deleted KCs failed to activate HSCs, indicating a contributory role of AEG-1 across multiple cell types in fibrogenic response [107].

Multiple studies have characterized SND1’s role in regulating TGF-β signaling, though the mechanistic directionality differs importantly between cancer contexts and warrants careful distinction [114,115,116]. In HCC cells specifically, SND1 functions as a post-transcriptional regulator that binds and stabilizes the mRNA of angiotensin II type 1 receptor (AT1R), promoting its translation; elevated AT1R surface expression subsequently activates SMAD2 phosphorylation and downstream TGF-β signaling, contributing to epithelial–mesenchymal transition (EMT) [114]. A mechanistically distinct relationship has been characterized in breast cancer cells, where TGF-β-induced SMAD activation directly transcribes the SND1 gene, and SND1 in turn recruits the histone acetyl transferase KAT2A/GCN5 to the promoter regions of SMAD2, SMAD3, and SMAD4, establishing a positive-feedback transcriptional amplification loop [115,116]. These two mechanisms—SND1 driving TGF-β signaling via AT1R mRNA stabilization in HCC versus TGF-β/SMADs driving SND1 transcription with reciprocal SMAD amplification in breast cancer—are not contradictory but represent context-specific regulatory relationships operating in distinct cell types and oncogenic settings [114,115,116]. Critically, whether the SMAD→SND1→SMAD amplification circuit operates in HCC-associated hepatic stellate cells or MASH-HCC hepatocytes has not been formally demonstrated, representing an important gap for future MASH-specific in vivo validation. Collectively, the available evidence positions both AEG-1 and SND1 as candidate regulatory nodes within TGF-β1-driven fibrogenic and EMT phenotypes in HCC, with the important caveat that their precise mechanistic contributions in the MASH-specific context require direct in vivo validation before definitive causal conclusions can be drawn [68,71,113,114,115,116].

### 3.3. Crosstalk and Immunosuppression

As HCC progresses from a pre-neoplastic nodule to established malignancy, the tumor microenvironment undergoes a fundamental immunological shift from the pro-inflammatory innate immune state toward an immunosuppressive niche that shields tumor cells from immune clearance. A key aspect of this switch is the reprogramming of tumor-associated macrophages (TAMs) from the classically activated, IL-12 producing, antigen-presenting, anti-tumor cytotoxic M1 phenotype to the alternatively activated, immune-tolerizing, angiogenic, and tumor-promoting M2 phenotype [21,117]. This polarization is driven by tumor-derived cytokines including IL-4, IL-10, IL-13, and VEGF, which collectively suppress M1 effector functions and upregulate M2 markers including CD163, mannose receptor C-type 1 (MRC1/CD206), IL-10, and arginase-1 in TAMs [21,117]. M2-polarized TAMs in turn upregulate the checkpoint ligand PD-L1 on both their own surface and on adjacent tumor cells through paracrine IL-10 and IL-6/STAT3 signaling, delivering persistent inhibitory signals to CD8^+^ T cells and driving them toward a state of functional exhaustion defined by co-expression of PD-1, hepatitis A virus cellular receptor 2 (HAVCR2/TIM-3), lymphocyte activating 3 (LAG3), and CTLA-4 [117,118]. Beyond the PD-1/PD-L1 axis, emerging evidence implicates IL-17 family signaling as an additional immunosuppressive circuit in the HCC tumor microenvironment, where IL-17A drives STAT3-dependent PD-L1 induction and stromal remodeling through hepatic stellate cell activation, representing a mechanistically distinct but complementary immunosuppressive axis alongside AEG-1/SND1-directed pathways [119].

AEG-1 is essential for effective macrophage function such as cell migration and clearing dead tissue [88]. However, in the context of an existing tumor, AEG-1 shifts its function to actively suppress the immune response. By driving the production of C-C motif chemokine ligand 3 (CCL3), C-X-C motif chemokine ligand 10 (CXCL10) and other chemical attractants in tumor cells, AEG-1 recruits circulating white blood cells into the tumor environment, where they are reprogrammed into tumor-protecting macrophages [71]. In addition, AEG-1 induces PD-L1 expression on HCC cells, via β-catenin/LEF-1 activation, to escape immune recognition and maintain T cell exhaustion [120]. Indeed, AEG-1 levels positively correlated with PD-L1 levels and negatively correlated with CD8^+^ T cells in HCC tissues from 391 patients [120]. This shows a double role of AEG-1, mediating the initial inflammatory injury that induces cancer development but then switching to an immunosuppressive environment that protects the mature tumor from immune attack.

SND1 also extends its immunosuppressive reach beyond the cancer cell itself. Melanoma cells release small transport vesicles (exosomes) loaded with SND1 which facilitates lung metastasis accompanied by increased TAM infiltration [121]. SND1 facilitated incorporation of CD47 into melanoma-derived exosomes allowing them to evade macrophage-mediated phagocytosis by inducing the ‘don’t eat me’ signal [121]. Knocking out SND1 facilitated activation of macrophages and type I T cell-mediated immunity against the melanoma cells [121]. This indicates that SND1 is protecting the tumor in two different ways. Internally by turning off protective genes inside the tumor cell, and externally by using these secreted exosomes to reprogram the surrounding immune cells. It remains to be seen whether exosomal SND1 exerts a similar effect in HCC as well. Together, AEG-1 and SND1 therefore operate through complementary intracellular and extracellular mechanisms to consolidate immune evasion AEG-1 by inducing PD-L1 and recruiting monocytes, and SND1 by packaging immunosuppressive load into exosomes that reprogram the broader tumor microenvironment.

In HCC, extracellular vesicles (EVs), such as exosomes, are important delivery mechanisms that spread signals to promote tumor growth, scarring, and immune evasion throughout the tumor environment [122]. Tumor-derived exosomes promote immune evasion by reprogramming TAMs toward an M2-like immunosuppressive phenotype and packaging PD-L1 to paralyze CD8^+^ T cells at a distance [123,124,125]. HSC-derived EVs further propagate fibrosis through paracrine metabolic reprogramming of adjacent hepatocytes. The metabolic stress of MASH influences exosome cargo loading, providing a mechanistic link between lipotoxic injury and immune suppression within the HCC microenvironment [126,127,128]. This vesicle-mediated immunosuppressive network, in which SND1 and AEG-1 have been implicated, may contribute to the limited 15–20% response rate observed with current immune checkpoint inhibitors, and highlights the need for therapeutic strategies that simultaneously disrupt oncogenic exosome biogenesis alongside conventional immune checkpoint blockade.

## 4. Molecular Carcinogenesis: The Engine of HCC

### 4.1. Oncogenes and Oncogenic Signaling Pathways

There are several oncogenic pathways that are most highly dysregulated among HCC patients, including the Wnt/β-catenin and the PI3K/Akt/mTOR signaling pathways, which act as central nodes in HCC integrating metabolic and inflammatory cues.

The Wnt/β-catenin pathway is among the most frequently dysregulated signaling axes in HCC patients [129]. Under conditions without Wnt ligands, cytosolic β-catenin associates in a complex with adenomatous polyposis coli (APC) and AXIN1 proteins, resulting in β-catenin phosphorylation [130]. This leads to ubiquitination and subsequent proteasomal degradation of β-catenin via the E3 ubiquitin ligase β-transducin repeat-containing protein (β-TRCP) [131]. When Wnt levels accumulate, the ligand binds to Frizzled receptors, preventing complex formation and subsequent β-catenin degradation. β-catenin is therefore able to translocate to the nucleus where it binds to T cell factor (TCF) and lymphoid enhancer-binding protein family (LEF) transcription factors to regulate cell proliferation and survival [130,131]. Under regulated conditions, Wnt/β-catenin signaling is vital for liver zonation, as indicated through a periportal phenotype in mice with an inducible loss of β-catenin [132]. In addition to its role in liver zonation, Wnt/β-catenin also plays an important role in liver regeneration, hepatotoxicity, hypoxia, and protection from hepatic steatosis [133,134,135,136].

Aberrant activation of β-catenin signaling promotes hepatocyte proliferation, cellular dedifferentiation, and the acquisition of stem-like properties that promote tumor initiation and progression [137,138,139]. Mutation and subsequent activation of the Wnt/β-catenin pathway is observed in 20–35% of HCC cases, making it the second most frequent mutation among patients [129,140]. The most common mutations occur at the serine/threonine sites of exon 3 in CTNNB1, which prevent β-catenin phosphorylation and subsequent proteasomal degradation [141]. Mutations in APC and AXIN1 have also been found in 3% and 8% of HCC cases, respectively [141,142,143]. Although Wnt/β-catenin mutations are insufficient to promote HCC in isolation in mouse models, β-catenin may cooperate with other oncogenes, such as H-Ras, to promote HCC [144,145,146,147].

In parallel, the PI3K/Akt/mTOR pathway serves as a central mediator of growth factor and nutrient signaling, linking metabolic dysregulation to oncogenic transformation in HCC [148]. Activation of PI3K/Akt signaling relies on both extracellular signals such as insulin, EGF, and IGF1, and intracellular signals such as nutrients and energy status [149]. The pathway is regulated by the key negative regulator PTEN, which is highly sensitive to inactivation by ROS [150,151]. In the liver, hyperinsulinemia and increased circulating lipids provide consistent stimuli that activate Akt independent of oncogenic mutations. Once activated, phosphorylated Akt reprograms cellular metabolism through mammalian target of rapamycin complex 1 (mTORC1) activation, and protein synthesis and cellular growth are enhanced in parallel with increased glycolytic flux, a hallmark of the Warburg Effect. Simultaneously, Akt drives lipogenesis through upregulation of sterol regulatory element-binding proteins (SREBPs) and FASN, providing biosynthetic substrates required for further proliferation [152]. Akt-mediated inhibition of GSK3β can further stabilize β-catenin, linking metabolic reprogramming to the proliferative Wnt-driven transcriptional program in HCC.

Transcriptome analysis of 603 patients allowed stratification of HCC patients into three subclasses, S1, S2 and S3 [153]. The S1 subclass is marked by aberrant activation of WNT signaling, the characteristic feature of the S2 subclass is MYC and AKT activation, and S3 was associated with more differentiated phenotype with better prognosis [153]. MYC, coding for the transcription factor c-Myc, is frequently amplified in HCC patients, and c-Myc overexpression has been observed in 30–70% HCC patients [154,155,156]. MYC overexpression alone can induce HCC in mice, indicating that MYC functions as a driver oncogene, and the oncogenic function of MYC depends upon downstream mTORC1-AKT activity [156,157]. Additional oncogenes that are amplified in HCC include cyclin D1 (CCND1), FGF3, FGF4 and FGF19 [129]. There is strong cooperation between AEG-1 and MYC. Both genes reside in chromosome 8q and are co-amplified in HCC patients [68]. c-Myc binds directly to AEG-1 promoter inducing its transcription [44]. AEG-1 interacts with the transcription repressor zinc finger and BTB domain containing 16 (ZBTB16/PLZF) and neutralizes it allowing induction of MYC transcription [158]. AEG-1 also induces MYC expression by activating Wnt/β-catenin signaling pathway [43]. Functionally, AEG-1 and c-Myc cooperate, so that overexpression of both genes in mouse liver induces spontaneous, metastatic HCC, which is not observed upon overexpression of each gene alone [68]. Together, AEG-1 and c-Myc induce a unique gene signature of non-coding RNAs (ncRNAs) facilitating aggressive hepatocarcinogenesis [68].

### 4.2. The RNA-Induced Silencing Complex (RISC) and Gene Silencing

**Figure 3:**
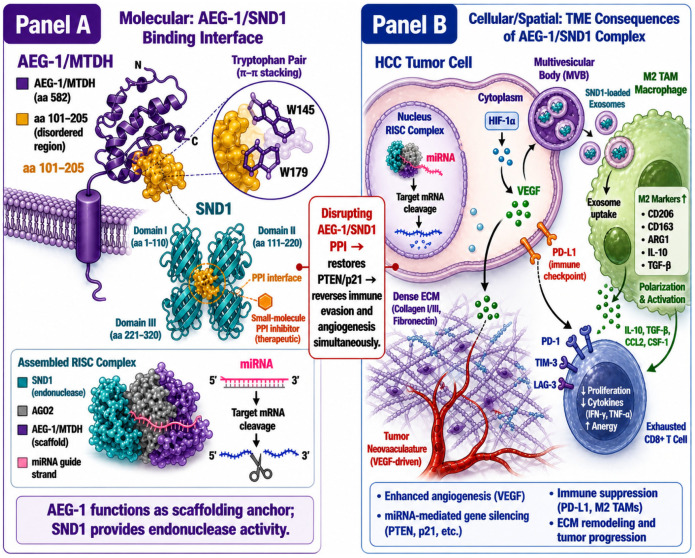
Molecular structure and microenvironmental consequences of the astrocyte elevated gene-1 (AEG-1)/staphylococcal nuclease and tudor domain-containing 1 (SND1) complex. (**Panel A**) Molecular Interface: AEG-1 functions as a crucial scaffolding anchor via its disordered region (amino acids 101–205). It physically binds the endonuclease SND1, stabilized by tryptophan stacking (W145/W179) to assemble the fully active RNA-induced silencing complex (RISC) alongside Argonaute 2 (AGO2). (**Panel B**) Tumor microenvironment (TME) Consequences: Within the hepatocellular carcinoma (HCC) cell, this hyperactive RISC mediates the cleavage of target messenger RNAs (mRNAs), silencing critical tumor suppressors (e.g., phosphatase and tensin homolog (PTEN), cyclin-dependent kinase inhibitor 1A (p21)). Concurrently, this axis drives a highly aggressive TME through three pathways: (1) sustaining hypoxia-inducible factor 1-alpha (HIF-1α)/vascular endothelial growth factor (VEGF) signaling to promote dense tumor neovasculature; (2) secreting SND1-loaded exosomes that polarize nearby macrophages into an immunosuppressive M2 phenotype (characterized by cluster of differentiation 206 (CD206), cluster of differentiation 163 (CD163), arginase 1 (ARG1), and interleukin 10 (IL-10)); and (3) upregulating surface programmed death-ligand 1 (PD-L1), which binds programmed cell death protein 1 (PD-1) on cluster of differentiation 8-positive (CD8^+^) T cells to trigger severe functional exhaustion and anergy. Therapeutic Disruption: As highlighted here, targeted small-molecule inhibitors designed to break the AEG-1/SND1 protein–protein interaction (PPI) represent a potential strategy to simultaneously restore tumor suppression, halt angiogenesis, and reverse immune evasion. Schematic prepared by the authors. Visualization was created with the assistance of Perplexity.ai (https://www.perplexity.ai/, accessed on 25 May 2006).

Although canonical gene mutations constitute a large portion of the drivers of oncogenesis in HCC, post-transcriptional modifications driven through activation of the RNA-induced silencing complex (RISC) also play a role in HCC onset and progression. RISC is a multiprotein complex that mediates miRNA-guided gene silencing by targeting complementary messenger RNAs (mRNAs) for degradation, thereby modulating gene expression [49]. SND1 is a core component of the RISC with endonuclease activity that allows for RNA interference (RNAi)-mediated gene silencing [49]. AEG-1 interacts with SND1 as well as with argonaute RISC catalytic component 2 (AGO2) and both AEG-1 and SND1 contributed to RISC activity in HCC cells (Figure 3) [50]. Increased RISC activity, contributed by overexpressed AEG-1 or SND1, resulted in decreased expression of tumor suppressor genes that are regulated by miRNAs, such as PTEN and CDKN1A/p21 [50].

### 4.3. The Angiogenic Switch

A defining characteristic of HCC progression is the “angiogenic switch”, which enables tumor cells to proliferate beyond the limits of normal nutrient diffusion via the expression of angiogenic factors to promote vascularization and enhanced growth [159]. AEG-1 has been shown to promote angiogenesis in HCC. Vascular endothelial growth factor (VEGF), which drives angiogenesis in HCC, was shown to significantly increase in AEG-1 overexpressing HepG3 xenograft models, resulting in highly vascular tumors [43]. Overexpression of AEG-1 in mouse hepatocytes also exerts a strong angiogenic response [67,68]. Angiopoietins and chemokines are also regulated by AEG-1 [160]. AEG-1 has also been shown to stabilize HIF-1α and enhance its transcriptional activity, establishing a feed-forward loop whereby hypoxia and oncogenic signaling cooperate to amplify AEG-1 and VEGF expression and angiogenic progression [161,162,163,164].

SND1 promotes tumor angiogenesis in HCC through a signaling cascade characterized primarily in HCC cell lines that operates in parallel to AEG-1-mediated VEGF induction. Overexpressed SND1 activates NF-κB, which directly transactivates the oncomiR miR-221 [165]. miR-221 in turn induces the expression of two potent angiogenic mediators: Angiogenin, a ribonuclease that stimulates endothelial cell proliferation, tube formation, and neovascularization; and CXCL16, a chemokine that recruits pro-angiogenic immune cells into the tumor microenvironment [165]. This cascade was functionally characterized in HCC cell lines, where conditioned medium from SND1-overexpressing Hep3B cells significantly augmented angiogenesis in chorioallantoic membrane assays and HUVEC tube formation assays, while stable SND1 knockdown markedly suppressed these angiogenic responses [165]. It is important to acknowledge, however, that direct in vivo validation of this SND1/NF-κB/miR-221 axis specifically within HCC-associated vascular endothelial cells remains to be demonstrated, and its interaction with VEGF-dependent therapeutic targets such as Sorafenib and Bevacizumab represents an important open question for future investigation. Beyond this pathway, SND1 has also been shown to regulate the SND1/hTERT axis, through which sodium butyrate simultaneously suppresses SND1 to exert pro-apoptotic and anti-angiogenic effects in HCC cells, suggesting additional mechanisms by which SND1 may contribute to the HCC angiogenic program [166].

Hypoxia represents a critical upstream driver of angiogenesis through stabilization of the transcription factor hypoxia-inducible factor-1α (HIF-1α) [167]. Under normoxic conditions, HIF-1α is rapidly degraded; however, under the hypoxic conditions of the tumor microenvironment, HIF-1α accumulates and activates a cascade of signaling pathways involved in metabolism, survival, and angiogenesis [167]. The resulting hypoxic microenvironment further reinforces both SND1 and AEG-1 expression, suggesting that both oncoproteins are not only upstream regulators but also downstream effectors of hypoxic signaling, creating compounding feed-forward angiogenic loops [161,162,163,164].

Beyond transcriptional regulation, the angiogenic switch involves remodeling of tumor vasculature. Newly formed vessels are often structurally abnormal, characterized by irregular branching, high permeability, and poor perfusion [168]. These features contribute to the hypoxic conditions that reinforce the HIF-1α/AEG-1/SND1 feed-forward loop. Simultaneously, tumor-derived factors stimulate endothelial cell proliferation and tube formation, while also recruiting pericytes and stromal components that support vessel stabilization and growth, features which are also augmented by both AEG-1 and SND1 [159,169].

## 5. Therapeutic Implications and Translational Opportunities

Liver cancer is not driven by a single factor but is the result of a complex web of metabolic damage, inflammation, scarring and unregulated cell growth. This complexity explains why treatments relying on a single drug consistently fail to provide long-term benefits for patients with advanced disease. Understanding why current treatments fail and identifying the specific weaknesses they miss provide the foundation for a new generation of therapies designed to attack the exact root causes of HCC.

### 5.1. Mechanisms of Resistance: Why Sorafenib Fails: Adoptive Angiogenesis and Vessel Co-Option

Sorafenib is a multi-target tyrosine kinase inhibitor (TKI) that blocks VEGFR2, PDGFR, and Raf kinases. It was the first systemic therapy approved for advanced HCC and remains a frontline standard of care [170]. Its primary mechanism of action is anti-angiogenic, by blocking VEGFR signaling it prevents the blood vessel formation on which cancer depends on for growth. However, clinical benefit is modest, median overall survival is extended by approximately 2–3 months, compared to placebo, and nearly all patients who initially respond develop resistance [34,171].

Two mechanisms are principally responsible for sorafenib resistance, the first of which is adaptive angiogenesis. In response to VEGFR blockade, hypoxic tumor cells cause a compensatory transcriptional response mediated by HIF-1α and HIF-2α, which upregulates alternative pro-angiogenic mediators, including fibroblast growth factor (FGF), placental growth factor (PlGF) and Ang-2, effectively bypassing the VEGF blockade to maintain vascularization [172]. Also, sorafenib-mediated suppression of HIF-1α synthesis through Akt/mTOR and Raf/MEK/ERK inhibition triggers a compensatory upregulation of HIF-2α, which in turn activates the (TGF-α/EGFR) pathway and drives resistance in hypoxic tumor cells [172]. AEG-1 directly amplifies this escape. AEG-1 induces HIF-1α and enhances its transcriptional activity, maintaining VEGF and angiopoietin-1 expression under anti-VEGFR pressure providing a mechanistic support for AEG-1’s direct contribution to adaptive angiogenic resistance [160,161].

The second mechanism is vessel co-option. Rather than inducing new vessel formation, resistant tumor cells physically migrate toward and co-opt pre-existing vessels (sinusoidal and portal), bypassing the need for angiogenesis entirely [173,174]. Tumors undergoing this transition shift from relying on sprouting angiogenesis to depending almost entirely on co-opted pre-existing vessels [174]. This mechanism accounts for up to 75% of the total tumor vascularization in sorafenib-resistant HCC xenografts, compared to just 23% in untreated controls [174]. Gene signature analysis documented that vessel co-option is linked to EMT that AEG-1 promotes through PI3K/Akt and NF-κB activation, increasing tumor cell invasiveness and facilitating sinusoidal vessel infiltration in the face of angiogenic blockade [174,175]. Together, adaptive angiogenesis and vessel co-option represent a coordinated vascular escape program that makes anti-VEGFR therapy increasingly ineffective.

Beyond vascular escape, sorafenib resistance is compounded by compensatory activation of PI3K/Akt/mTOR and Wnt/β-catenin signaling the pathways which sustain tumor cell survival despite Raf/MEK/ERK blockade [33,35,176]. Long-term sorafenib treatment selectively enriches cancer stem cell (CSC) populations that activate these pathways, driving tumor recurrence with a more aggressive and drug-resistant phenotype [177,178].

Another major axis of resistance operates at the level of drug efflux. Multidrug resistance (MDR) transporters [ATP binding cassette subfamily B member 1/P-glycoprotein (ABCB1/P-gp), ATP binding cassette subfamily C member 1 (ABCC1/MRP1), and ATP binding cassette subfamily G member 2 (ABCG2)] are ATP-dependent membrane pumps that actively export chemotherapeutic agents from tumor cells before they can exert cytotoxic effects [179]. Their transcription is upregulated by NF-κB (the same pathway amplified by AEG-1 and SND1) creating a direct mechanistic link between AEG-1/SND1-driven inflammation and the acquirement of multidrug resistance [180,181,182]. AEG-1 binds to ABCB1 mRNA, increases its translation and also inhibits its degradation, thereby directly contributing to chemoresistance [182].

This connection was directly indicated by showing that AEG-1 activates the PI3K/Akt/HIF-1α/MDR-1 axis specifically under hypoxic conditions in HCC [161]. AEG-1 knockdown results in dramatic reductions in PI3K, Akt, HIF-1α, and MDR-1 protein expression alongside increased PTEN levels, supporting a role for AEG-1 as an upstream regulator of hypoxia-induced chemoresistance in HCC [161]. Collectively, these observations offer a plausible molecular rationale for the aggressive angiogenic phenotype and chemoresistance of hypoxic, AEG-1-overexpressing tumors. Consequently, AEG-1 emerges as a high-priority therapeutic target, as its inhibition might simultaneously attenuate both vascular escape and chemoresistance pathways.

### 5.2. Novel Molecular Targets

#### 5.2.1. Targeting the AEG-1/SND1 Axis: Disrupting Protein–Protein Interactions

The mechanistic framework of this review positions AEG-1 as a principal upstream driver, and SND1 as a cooperating partner, in the metabolic, inflammatory, oncogenic, and immunosuppressive networks that drive HCC. This positions the AEG-1/SND1 complex as a mechanistically attractive therapeutic target. The challenge is that AEG-1 is not an enzyme with a defined catalytic pocket, it is a scaffold protein that operates primarily through protein–protein interactions (PPIs). Therapeutic disruption of PPIs has historically been difficult, but the molecular characterization of AEG-1’s key binding interfaces now opens the door for several rational strategies. Among these, we contrast two structurally defined AEG-1 interfaces with opposite therapeutic implications: the SND1-binding groove, whose disruption removes oncogenic function, and the LXXLL/PPARα motif, whose disruption relieves one pathogenic function while releasing others. This contrast establishes that structural definition of an AEG-1 interaction is not, by itself, sufficient grounds for therapeutic targeting.

Analysis of crystal structure of minimal regions of AEG-1/SND1 interaction identified an 11 amino acid (a.a.) peptide in AEG-1 protein (DWNAPAEEWGN) residing between a.a. 393–403 [183]. Mutational analysis unraveled W394 and W401 as key residues in AEG-1 protein that mediate interaction with an extended protein groove between two SN domains (SN1/2) of SND1 [183]. Disrupting this interface through peptidomimetics, stabilized peptides, or small molecules might be efficient strategies to block oncogenic functions of AEG-1 and SND1. Indeed, peptides targeting this interaction have shown efficacy in breast cancer [184,185,186]. At the same time, small molecule inhibitors disrupting AEG-1/SND1 interaction inhibited breast cancer and synergized with anti-PD-1 immunotherapy [187,188,189].

A cautionary example illustrating the risk of overinterpreting mechanistic interactions as therapeutic targets is AEG-1’s LXXLL nuclear receptor-binding motif, through which AEG-1 physically engages and inhibits PPARα [71,73]. Although this interface is structurally well-defined, available in vivo data show that motif-specific disruption produces complex, context-dependent effects rather than the straightforward therapeutic benefit that PPARα-directed strategies might predict [74]. We include this example specifically to caution against assuming that any validated AEG-1 protein–protein interaction is automatically druggable in MASH-HCC; the LXXLL interface remains an informative feature of AEG-1 biology, not a standalone therapeutic target. On this basis we reassess AEG-1/PPARα as a therapeutic pairing and do not advance the LXXLL motif as a viable direct target in MASH-HCC: pharmacologic disruption of this interface would be predicted to relieve PPARα inhibition only at the cost of unleashing AEG-1’s lipogenic, mitogenic, and pro-inflammatory outputs, as observed in the AEG-1-L24K/L25H knock-in liver [74]. A plausible explanation is that in AEG-1-L24K/L25H cells, when AEG-1 does not interact with PPARα, more AEG-1 is available to interact with its other interacting proteins and RNAs. Indeed, RNA immunoprecipitation assay showed that AEG-1-L24K/L25H binds more to FASN mRNA compared to WT AEG-1, which might lead to an increase in de novo lipogenesis that may compensate for decreased fatty oxidation β-oxidation because of disruption of AEG-1/PPARα interaction [74]. This reassessment reinforces the central argument of this review—that therapeutic value lies in the SND1-binding groove, whose disruption removes oncogenic function without this trade-off, rather than in the LXXLL/PPARα motif.

A second consideration in therapeutic targeting of AEG-1 is its physiologically necessary role within the innate immune NF-κB activation complex. As described in Section 3.1, AEG-1 associates with K63-ubiquitinated TRAF2 and RIPK1 at the membrane-proximal signaling complex, stabilizing IKK activation and sustaining NF-κB-driven transcription of pro-inflammatory and pro-survival genes [95,96,97,99]. While this function is exploited oncogenically in HCC, TRAF2 and RIPK1 are equally essential mediators of physiological innate immune responses to pathogen-associated molecular patterns and death receptor signaling in non-malignant tissues [190,191]. Systemic or non-selective disruption of AEG-1’s interaction with TRAF2 or RIPK1 therefore carries a theoretical on-target risk of impairing innate immune surveillance, potentially rendering treated patients susceptible to opportunistic infections or dysregulated inflammatory responses a concern that is particularly relevant in the cirrhotic, immunocompromised MASH-HCC patient population. To date, no study has specifically attempted to disrupt the AEG-1–TRAF2 or AEG-1–RIPK1 protein–protein interaction as a therapeutic strategy, and whether selective disruption of these interfaces in malignant versus non-malignant cells is achievable remains an open question [192]. Hepatocyte- and Kupffer cell-targeted nanoparticle delivery platforms described in Section 5.3 offer a rational solution to this challenge—by confining AEG-1 silencing to specific compartments systemic NF-κB suppression in non-hepatic immune cells can be avoided, substantially mitigating the off-target immunotoxicity risk. Cell-type-selective delivery may therefore represent not only a technical advantage but a safety requirement for the clinical translation of AEG-1-targeted therapeutics.

At the gene expression level, siRNA-based silencing of AEG-1 has shown preclinical efficacy in HCC models [193]. Hepatocyte-targeted polyamidoamine (PAMAM) dendrimer-delivered AEG-1 siRNA in combination with all-trans retinoic acid (ATRA) produced profound synergistic inhibition of orthotopic human HCC xenografts in nude mice with marked reduction in tumor burden, suppression of AEG-1 mRNA, and restoration of retinoic acid-induced differentiation programs [193]. This study supported the therapeutic potential of nanoparticle-mediated AEG-1 siRNA delivery to the liver and identified the AEG-1/ATRA combination as a mechanistically grounded therapeutic strategy for HCC [193]. The primary remaining barrier to clinical translation of siRNA-based AEG-1/SND1 targeting is delivery which will be discussed in the next section. In parallel, locked nucleic acid-modified (LNA) antisense oligonucleotides (ASO) for AEG-1 has shown efficacy in colorectal and lung cancers [194]. AEG-1 LNA ASO sensitized HCC tumors to anti-PD-1 immunotherapy with accompanied infiltration of cytotoxic T cells [120].

#### 5.2.2. Metabolic Inhibitors Combined with Oncogene Targeting

The metabolic reprogramming driven by AEG-1 particularly its enhancement of FASN-dependent de novo lipogenesis and suppression of PPARα-mediated β-oxidation creates therapeutic vulnerabilities that can be targeted pharmacologically [71]. FASN inhibitors directly target the lipogenic enzyme whose translation AEG-1 promotes through preferential polysome association of FASN mRNA [71]. The first-in-class oral FASN inhibitor TVB-2640 has indicated significant preclinical and clinical activity. In the phase 2 FASCINATE-1 trial in MASH patients, TVB-2640 at 50 mg reduced liver fat by 28.1% compared to placebo, with dose-dependent improvements in inflammatory and fibrotic biomarkers, while preclinical evaluation of the related compound TVB-3664 indicated significant anti-tumor efficacy in HCC models establishing proof of concept for FASN inhibition as an anti-HCC strategy [195,196]. FASN inhibition may also suppress the lipotoxic lipid intermediate accumulation that drives the DAMP-mediated KC activation, potentially reducing the oncogenic inflammatory milieu independent of direct tumor cell targeting.

A landmark development in MASH-directed therapy is the FDA approval of resmetirom (Rezdiffra) in 2024 as the first pharmacotherapy specifically approved for non-cirrhotic MASH with moderate-to-advanced fibrosis (F2–F3) [197]. Resmetirom is a liver-directed, thyroid hormone receptor-β (THR-β) selective agonist that reduces hepatic lipid accumulation by stimulating mitochondrial fatty acid β-oxidation, suppressing de novo lipogenesis, and improving hepatic insulin sensitivity. In the phase III MAESTRO-NASH trial, resmetirom achieved MASH resolution without worsening fibrosis in 29.9% and 25.9% of patients at the 100 mg and 80 mg doses, respectively, compared to 9.7% in the placebo group [197]. THR-β uses RXR as its heterodimer partner, and AEG-1’s ability to inhibit RXR function also propagates toward inhibition of THR-β function [198]. Therefore, THR-β activation by resmetirom directly counters the lipogenic programs driven by AEG-1. While resmetirom has not yet been evaluated in established HCC, its capacity to interrupt the lipotoxic-inflammatory cascade that drives MASLD-to-MASH-to-HCC progression positions it as a potentially important upstream pharmacological intervention in MASH-HCC prevention. Whether resmetirom-mediated metabolic correction translates to reduced AEG-1/SND1 oncogenic signaling represents an important and currently unexplored translational question warranting future investigation.

Combination strategies targeting both the metabolic and oncogenic arms simultaneously are suggested by the mechanistic molecular networks described throughout this review. The PI3K/Akt/mTOR pathway not only drives the Warburg metabolic phenotype and lipogenesis, but also through Akt-mediated GSK3β inhibition stabilizes β-catenin and activates Wnt-driven transcriptional programs. In HCC, AEG-1 has been shown to interact with protein arginine methyltransferase 5 (PRMT5) contributing to activation of Wnt/β-catenin pathway [199]. Simultaneous inhibition of PI3K/mTOR and FASN might therefore suppress metabolic reprogramming, oncogenic β-catenin signaling, and the lipotoxic DAMP burden driving innate immune activation addressing three interconnected nodes of HCC pathogenesis in a single combination. Restoration of PPARα activity through fibrate-class agonists provides an additional complementary strategy. Fibrates would restore hepatic fatty acid oxidation, and reduce lipid intermediate accumulation; however, as discussed in Section 5.2, the in vivo consequences of disrupting the AEG-1–PPARα interface are not straightforward, and this strategy should be regarded as hypothesis-generating.

### 5.3. Advanced Delivery Systems: Nanomedicine

The liver presents both an opportunity and a paradox for drug delivery. On one hand, it is highly accessible to circulating molecules via its fenestrated sinusoidal endothelium and receives approximately 25% of cardiac output [200]. On the other hand, achieving selective delivery to a specific cell type (hepatocytes, KCs, or HSCs) within this heterogeneous tissue is technically challenging. Systemically administered small molecules distribute broadly, producing off-target toxicity that limits achievable therapeutic doses. siRNA and ASO therapeutics, despite their molecular specificity, are rapidly degraded by serum nucleases, cleared by the kidneys, and poorly internalized by target cells without a delivery vehicle [201]. The liver non-parenchymal cells (KCs and HSCs) do not express the asialoglycoprotein receptor (ASGPR) that mediates hepatocyte-selective uptake, therefore requiring fundamentally different delivery strategies for each cellular compartment [201]. Lipid nanoparticles (LNPs) have emerged as the dominant platform for hepatic siRNA delivery, with clinical validation exemplified by the FDA approval of Patisiran (Onpattro), the first FDA-approved siRNA therapeutic, which uses ionizable LNPs to deliver siRNA to hepatocytes via ASGPR-mediated endocytosis [202]. The core concept is encapsulation of siRNA within an ionizable lipid nanoparticle that is cationic at low endosomal pH (for endosomal escape and cytoplasmic siRNA release) but neutral at physiological pH (to avoid off-target interactions and immune activation) [202]. Surface functionalization with GalNAc (N-acetylgalactosamine) ligands also improves ASGPR-mediated hepatocyte targeting with almost complete hepatocyte specificity and significantly reduced toxicity at clinically relevant doses [203]. For HCC specifically, LNP delivery of siRNA to hepatocytes has shown promising preclinical efficacy: LNP delivery of siRNA targeting JNK2 in murine HCC models led to significant reduction in hepatocyte apoptosis, decreased fibrogenesis and improved hepatic parenchymal architecture, supporting the utility of this platform for hepatocellular targets [204]. The therapeutic potential of PAMAM dendrimer-based nanoparticles for hepatocyte-targeted delivery of AEG-1 siRNA has been indicated in MASH and HCC [71,193]. PAMAM dendrimers are well-defined, branched polymeric nanoparticles with a controlled surface chemistry that allow for efficient complexation and intracellular delivery of siRNA [205]. Hepatocyte-targeted PAMAM-AEG-1 siRNA significantly reduced AEG-1 mRNA in orthotopic HCC xenografts, suppressed tumor burden, and restored expression of retinoic acid-responsive tumor suppressor genes when combined with ATRA, establishing a direct translational proof of concept for nanoparticle-mediated AEG-1 targeting in HCC [193]. Because intravenously administered nanoparticles distribute preferentially to the liver, HCC represents the tumor type most likely to derive immediate therapeutic benefit from systemic nanoparticle-siRNA delivery platforms [193].

For targeting KCs and M2 TAMS, whose AEG-1-driven immunosuppressive activity is a potential therapeutic target, nanoparticles functionalized with mannose receptor (CD206) ligands provide selective delivery to M2-polarized TAMs within the HCC TME [206]. Mannose-decorated liposomes loaded with AEG-1 siRNA might preferentially accumulate in M2 TAMs, delivering gene silencing specifically to the cell population where AEG-1 exerts its most consequential immunosuppressive function. Overall, these delivery strategies highlight the potential of AEG-1- and SND1-targeted therapy in HCC. Further work will be needed to define which delivery approaches provide the best balance of efficacy and specificity.

## 6. Conclusions

### 6.1. Limitations and Boundaries of the AEG-1/SND1 Model

The mechanistic framework presented in this review positions the AEG-1/SND1 axis as a convergent node linking metabolic injury, inflammation, immune evasion, and therapeutic resistance in MASH-HCC. However, several important boundaries of this model warrant explicit acknowledgment, especially the role of SND1. It is important to recognize that much of the evidence positioning AEG-1 and SND1 as convergent oncogenic nodes in MASH-HCC derives from gain- and loss-of-function studies in hepatocyte-derived cell lines and xenograft models, supplemented by correlative overexpression analyses in human HCC tissue. While these approaches establish mechanistic plausibility and consistent associative patterns across experimental systems, they do not constitute direct causal proof in the context of spontaneous, diet-induced MASH-HCC in immunocompetent hosts. Conditional hepatocyte-specific or myeloid cell-specific AEG-1 knockout mice show marked resistance to high-fat diet-induced MASH indicating that AEG-1 is necessary in the pathogenesis of MASH [71,107]. High-fat diet-fed conditional hepatocyte-specific SND1 knockout mice did not show any difference in phenotype when compared to their control littermates suggesting that SND1 loss alone is insufficient to reproduce AEG-1-dependent phenotype [82]. Whole body SND1 knockout mouse did not show any noticeable phenotype, except for a decrease in total myeloid cells [81]. However, overexpression of SND1 in hepatocytes caused an increase in the levels of cholesterol esters along with increased activity of SREBP2 [77,78,80]. Collectively, these studies suggest that AEG-1 is a major regulator of lipid metabolism and inflammation; whereas, SND1 might be involved in regulating myeloid cell function, hence inflammation, and it may regulate lipid metabolism only when overexpressed. Analysis of myeloid cell-specific SND1 knockout mouse as well as hepatocyte- or myeloid cell-specific AEG-1 overexpressing mouse with deletion of SND1 will provide in-depth mechanistic insight into the role of AEG-1/SND1 in MASH and MASH-HCC. Importantly, none of these studies followed these mice long-term so the relevance of these findings to progression from MASH to MASH-HCC remains unresolved. As such, the mechanistic relationships between AEG-1/SND1 axis and MASH-HCC described in this review should be understood as supported more by convergent preclinical evidence rather than by direct in vivo causal genetics.

There are some additional limitations. First, HCC is a molecularly heterogeneous disease, and the AEG-1/SND1-driven immunosuppressive model described here may be less applicable in the WNT/β-catenin-mutant HCC subclass—representing approximately 30–40% of cases—where immune exclusion is governed primarily by Wnt-mediated suppression of T cell homing rather than by macrophage polarization or checkpoint ligand upregulation. Whether AEG-1/SND1 contributes meaningfully to this immune-excluded phenotype in CTNNB1-mutant tumors remains unresolved.

Second, AEG-1’s role as a metabolic initiator of pre-neoplastic MASH injury is specific to the lipotoxic context of MASLD/MASH and should not be directly extrapolated to virally driven HCC, where the oncogenic landscape is shaped by distinct mechanisms including HBV insertional mutagenesis and HCV-mediated epigenetic reprogramming.

Third, a substantial proportion of the mechanistic data cited in this review derives from generic HCC cell lines—including HepG2, Hep3B, and SK-HEP-1—that do not recapitulate the steatotic, lipotoxic microenvironment of MASH. Findings from these systems should be interpreted with appropriate caution, and the Evidence Grading Table (Table 1) explicitly distinguishes cell line data from MASH-relevant in vivo validation throughout.

Finally, the immunosuppressive HCC tumor microenvironment encompasses mechanisms beyond the AEG-1/SND1 axis, including regulatory T cells (Treg) expansion, myeloid-derived suppressor cells (MDSC) accumulation, natural killer (NK) cell dysfunction, and IL-17 family signaling [119], none of which are fully explained by the model presented here. AEG-1/SND1 should therefore be understood as one mechanistically characterized and potentially actionable node within a broader immunosuppressive network.

### 6.2. HCC as a Disease of Metabolic-Inflammatory-Oncogenic Coupling

This review demonstrates that HCC does not arise from a single molecular insult but rather from a self-sustaining cycle in which metabolic dysfunction, chronic inflammation, and oncogenic transformation converge and amplify one another over years of progressive liver injury. Within this framework, the available evidence supports AEG-1 as an upstream driver that contributes to the initiation and propagation of the cycle, with SND1 acting as a cooperating partner in the oncogenic arm of the network.

AEG-1 initiates and sustains this cycle. In the pre-cancerous liver, AEG-1 drives fat accumulation by blocking the liver’s fat-burning machinery (PPARα) while simultaneously turning up fat production (FASN) [71]. It amplifies the NF-κB inflammatory response in both hepatocytes and Kupffer cells, prevents damaged hepatocytes from dying when they should, and reprograms immune cells into a tumor-friendly state [71,107]. AEG-1 also regulates high-fat diet-induced adipocyte inflammation [107], which is important for releasing free fatty acids (FFAs) from adipocytes into the circulation, and FFAs then accumulate in the liver as triglycerides. While SND1 activates NF-κB, SND1 itself is transcriptionally induced by NF-κB, thereby augmenting inflammation further [207]. Inflammation-induced overexpressed SND1 might contribute to MASH propagation by facilitating accumulation of cholesterol esters in hepatocytes [77]. In the established tumor, AEG-1 recruits SND1 to silence tumor suppressor genes through RISC, keeps the tumor’s blood supply growing by stabilizing HIF-1α, and spreads immunosuppressive signals to surrounding cells through extracellular vesicles [50]. Few proteins characterized in HCC have been implicated across a comparable breadth of disease compartments, which motivates continued interest in AEG-1 as an upstream target.

### 6.3. Future Directions: Single-Cell Profiling and Theranostics

Most of what we know about AEG-1, SND1, and the HCC tumor microenvironment comes from studying bulk tissue samples, that obscure the profound cellular heterogeneity of the hepatic tumor niche. Single-cell RNA sequencing and spatial transcriptomics now offer the resolution needed to map AEG-1/SND1-driven gene regulation at the level of individual cell populations within their architectural context, enabling identification of stage-specific therapeutic windows that bulk analyses cannot resolve [107,208,209,210,211].

Spatial transcriptomics is especially well-suited to HCC because it maps gene expression while preserving tissue architecture—meaning one can determine not just which cells express AEG-1 or SND1, but exactly where they sit relative to immune cell aggregates, fibrotic regions, and tumor margins within an intact tissue section. Recent work integrating scRNA-seq and spatial transcriptomics in HCC patient cohorts has already demonstrated that spatial organization of tumor-immune boundaries predicts immune exclusion and patient prognosis in ways that bulk transcriptomics cannot see. Importantly, early spatial transcriptomic studies from our laboratory in AEG-1 mouse liver models revealed palmitoylation-dependent and liver zonation-dependent patterns of AEG-1 gene regulation that were not detectable in bulk RNA analyses, underscoring the biological depth that remains to be uncovered [211]. These technical approaches also help unravel MASH regulation by myeloid cell AEG-1 [107]. Applying this technology to human MASH-HCC tissue, stratified by disease stage and therapeutic history, represents an immediate and high-priority research direction.

A second important direction is the development of theranostics—nanoparticle platforms engineered to simultaneously deliver AEG-1/SND1 siRNA and report on biodistribution and therapeutic response through integrated imaging agents offering a direct path toward real-time adaptive personalization of siRNA-based HCC therapy [212,213,214]. The multicentric and heterogeneous nature of HCC makes such real-time pharmacodynamic monitoring potentially valuable clinically, as tumor nodules within the same liver may respond differently to treatment in ways that are currently invisible and unmeasured in standard clinical practice. A proof-of-concept study using SP94-targeted nanospheres co-loaded with siRNA and iron oxide nanoparticles has indicated the feasibility of integrated diagnosis and gene therapy in preclinical HCC models. Extending this platform to AEG-1/SND1 siRNA represents a concrete translational opportunity.

Finally, the gut–liver axis deserves greater attention within the AEG-1/SND1 framework. The gut–liver axis—specifically the mechanism by which gut dysbiosis-derived TLR4 ligands modulate AEG-1-driven NF-κB inflammation—and whether microbiome-targeted interventions could blunt AEG-1-driven hepatocarcinogenesis represents an important and currently underexplored dimension of MASH-HCC pathogenesis that warrants dedicated future investigation.

### 6.4. Moving Beyond VEGF-Centric Therapies Toward Multi-Targeted Precision Medicine

The evidence assembled in this review supports the following interpretation: the modest and transient clinical benefits achieved by successive generations of VEGF-centric anti-angiogenic therapies in HCC are a predictable consequence of targeting a downstream output of a far larger and more interconnected oncogenic network. Blocking VEGF signaling commonly triggers adaptive angiogenic escape, vessel co-option, and cancer stem cell enrichment because the upstream drivers of these compensatory responses—including AEG-1-mediated HIF-1α stabilization and NF-κB-driven transcription—remain active. Sustained therapeutic benefit in MASH-HCC will require targeting the upstream molecular hubs that maintain the full pathogenic network rather than its individual downstream effectors.

AEG-1 and SND1 are precisely those hubs. Critically, the AEG-1/SND1 interaction interface has now been structurally characterized, with W394/W401 residues of AEG-1 and the SN1/SN2 groove of SND1 identified as essential binding determinants—providing concrete structural targets for rational small-molecule drug design [183]. Combined with hepatocyte- and macrophage-selective nanoparticle delivery platforms that restrict silencing to the hepatic tumor compartment while preserving systemic innate immune function, multi-node disruption of the AEG-1/SND1 axis is technically achievable. Future clinical translation should incorporate patient stratification by AEG-1/SND1 expression, TME immune polarization state, and MASH fibrosis stage, alongside theranostic delivery platforms that enable real-time target engagement monitoring. The convergence of structurally characterized molecular targets, advanced delivery technology, and single-cell spatial profiling tools creates an opportunity to move beyond the incremental survival benefits that have defined HCC therapy for two decades, toward a mechanistically informed precision medicine approach capable of disrupting the disease at its molecular foundation.

## Figures and Tables

**Table 1:** Hierarchical evidence grading for key mechanistic claims involving AEG-1 and SND1 in MASH-associated hepatocellular carcinoma.

Mechanistic Claim	Level I Human Tissue	Level II In Vitro HCC	Level III In Vivo MASH/HCC	Level IV Therapeutic Data
AEG-1 drives MASH initiation via PPARα suppression and FASN-mediated lipogenesis	+++	+++	+++	+
AEG-1 amplifies NF-κB-driven hepatic inflammation and innate immune activation	+++	+++	+++	+
AEG-1/SND1 complex mediates RISC-dependent silencing of tumor suppressors PTEN and p21	++	+++	++	++
SND1 promotes tumor angiogenesis via NF-κB/miR-221/Angiogenin–CXCL16 axis	−	+++	+	−
AEG-1 orchestrates tumor immunosuppression via PD-L1 upregulation and monocyte recruitment	+++	+++	++	+++

Evidence was assessed across four levels of experimental rigor: (I) human tissue-based association studies; (II) functional in vitro studies in HCC cell lines; (III) in vivo validation in MASH-relevant or HCC-specific animal models; and (IV) therapeutic interaction or translational data. Evidence strength is denoted as: +++ = strong, reproducible, direct evidence from multiple independent studies; ++ = moderate evidence from limited or indirect studies; + = preliminary or model-extrapolated evidence from a single study; − = no evidence currently available.

## Data Availability

No new data were created or analyzed in this study. Data sharing is not applicable to this article.

## Abbreviations

The following abbreviations are used in this manuscript:

AASLD	American Association for the Study of Liver Diseases
ABCB1	ATP-Binding Cassette Subfamily B Member 1 (P-glycoprotein/P-gp)
ABCC1	ATP-Binding Cassette Subfamily C Member 1 (MRP1)
ABCG2	ATP-Binding Cassette Subfamily G Member 2
AEG-1	Astrocyte Elevated Gene-1 (also known as Metadherin/MTDH)
AFP	Alpha-Fetoprotein
AGO2	Argonaute 2
ALK5	Activin Receptor-Like Kinase 5
Ang-1	Angiopoietin-1
Ang-2	Angiopoietin-2
APC	Adenomatous Polyposis Coli
ASGPR	Asialoglycoprotein Receptor
ASO	Antisense Oligonucleotide
ATF6	Activating Transcription Factor 6
ATP	Adenosine Triphosphate
ATRA	All-Trans Retinoic Acid
AXIN1	Axis Inhibition Protein 1
BCL-2	B-Cell Lymphoma 2
BIM	BCL-2-Like Protein 11 (pro-apoptotic)
BiP/GRP78	Binding Immunoglobulin Protein/Glucose-Regulated Protein 78
β-catenin	Beta-Catenin (CTNNB1 protein product)
β-oxidation	Beta-Oxidation (mitochondrial fatty acid oxidation pathway)
β-TRCP	Beta-Transducin Repeat-Containing Protein (E3 ubiquitin ligase)
c-Myc	Cellular Myelocytomatosis Proto-Oncogene
CBP	CREB-Binding Protein
CCL2	C-C Motif Chemokine Ligand 2
CD8^+^	Cluster of Differentiation 8 (cytotoxic T cells)
CD163	Cluster of Differentiation 163 (M2 macrophage marker)
CD206	Cluster of Differentiation 206 (Mannose Receptor)
CDKN1A	Cyclin-Dependent Kinase Inhibitor 1A (p21)
CHOP	C/EBP Homologous Protein
CI	Confidence Interval
CSC	Cancer Stem Cell
CTLA-4	Cytotoxic T-Lymphocyte-Associated Protein 4
CTNNB1	Catenin Beta-1 (β-catenin gene)
DAGs	Diacylglycerols
DAMPs	Damage-Associated Molecular Patterns
DEN	Diethylnitrosamine
dsDNA	Double-Stranded DNA
EASL	European Association for the Study of the Liver
ECM	Extracellular Matrix
EGF	Epidermal Growth Factor
EGFR	Epidermal Growth Factor Receptor
eIF2α	Eukaryotic Initiation Factor 2 Alpha
EMT	Epithelial-to-Mesenchymal Transition
ER	Endoplasmic Reticulum
ERAD	ER-Associated Degradation
EVs	Extracellular Vesicles
FAK	Focal Adhesion Kinase
FASN	Fatty Acid Synthase
FFA	Free Fatty Acid
FGF	Fibroblast Growth Factor
FSP1	Ferroptosis Suppressor Protein 1
GalNAc	N-Acetylgalactosamine
GPX4	Glutathione Peroxidase 4
GRP94	Glucose-Regulated Protein 94
GSH	Reduced Glutathione
GSK3β	Glycogen Synthase Kinase 3 Beta
HBV	Hepatitis B Virus
HCC	Hepatocellular Carcinoma
HCV	Hepatitis C Virus
HGF	Hepatocyte Growth Factor
HIF-1α	Hypoxia-Inducible Factor-1 Alpha
HIF-2α	Hypoxia-Inducible Factor-2 Alpha
HIV-1	Human Immunodeficiency Virus Type 1
HMGB1	High Mobility Group Box 1
HSCs	Hepatic Stellate Cells
IGF1	Insulin-Like Growth Factor 1
IKK	IκB Kinase
IKKβ	IκB Kinase Beta
IL-1β	Interleukin-1 Beta
IL-4	Interleukin-4
IL-6	Interleukin-6
IL-10	Interleukin-10
IL-12	Interleukin-12
IL-13	Interleukin-13
IRE1α	Inositol-Requiring Enzyme 1 Alpha
JNK	c-Jun N-Terminal Kinase
KASL	Korean Association for the Study of the Liver
KCs	Kupffer Cells
LAG-3	Lymphocyte Activation Gene-3
LEF	Lymphoid Enhancer-Binding Factor
LNPs	Lipid Nanoparticles
LPCs	Lysophosphatidylcholines
LPS	Lipopolysaccharide
LXXLL	Leucine-X-X-Leucine-Leucine (nuclear receptor-binding motif)
MAFLD	Metabolic Dysfunction-Associated Fatty Liver Disease
MASH	Metabolic Dysfunction-Associated Steatohepatitis
MASLD	Metabolic Dysfunction-Associated Steatotic Liver Disease
MDM2	Mouse Double Minute 2 Homolog
MDR	Multidrug Resistance
MEK	Mitogen-Activated Protein Kinase Kinase
miRNA	MicroRNA
MLKL	Mixed Lineage Kinase Domain-Like Pseudokinase
mRNA	Messenger RNA
MRI	Magnetic Resonance Imaging
MRP1	Multidrug Resistance-Associated Protein 1 (ABCC1)
MTDH	Metadherin (also known as AEG-1)
mTOR	Mechanistic Target of Rapamycin
MTORC1	mTOR Complex 1
NF-κB	Nuclear Factor Kappa-Light-Chain-Enhancer of Activated B Cells
NLRP3	NLR Family Pyrin Domain-Containing 3 (inflammasome)
p21	Cyclin-Dependent Kinase Inhibitor 1 (CDKN1A)
p53	Tumor Protein p53
PAMAM	Polyamidoamine (dendrimer)
PD-1	Programmed Cell Death Protein 1
PD-L1	Programmed Death-Ligand 1
PDGF-BB	Platelet-Derived Growth Factor BB
PDGFR	Platelet-Derived Growth Factor Receptor
PERK	Protein Kinase RNA-Like ER Kinase
P-gp	P-Glycoprotein (ABCB1)
PI3K	Phosphoinositide 3-Kinase
PlGF	Placental Growth Factor
PPARα	Peroxisome Proliferator-Activated Receptor alpha
PPIs	Protein–Protein Interactions
PTEN	Phosphatase and Tensin Homolog
PUMA	p53 Upregulated Modulator of Apoptosis
Raf	Rapidly Accelerated Fibrosarcoma Kinase
RIPK1	Receptor-Interacting Serine/Threonine-Protein Kinase 1
RIPK3	Receptor-Interacting Serine/Threonine-Protein Kinase 3
RISC	RNA-Induced Silencing Complex
RNAi	RNA Interference
ROS	Reactive Oxygen Species
scRNA-seq	Single-Cell RNA Sequencing
Shh	Sonic Hedgehog
siRNA	Small Interfering RNA
SLC7A11	Solute Carrier Family 7 Member 11 (system Xc^−^ transporter)
α-SMA	Alpha-Smooth Muscle Actin
SMAD2	SMAD Family Member 2
SMAD3	SMAD Family Member 3
SMAD4	SMAD Family Member 4
SND1	Staphylococcal Nuclease Domain-Containing 1
STAT3	Signal Transducer and Activator of Transcription 3
STING	Stimulator of Interferon Genes
TAMs	Tumor-Associated Macrophages
TCF	T Cell Factor
TGF-α	Transforming Growth Factor Alpha
TGF-β1	Transforming Growth Factor Beta 1
TGFβRII	Transforming Growth Factor Beta Receptor II
TIM-3	T Cell Immunoglobulin and Mucin Domain-Containing Protein 3
TIMPs	Tissue Inhibitors of Metalloproteinases
TKIs	Tyrosine Kinase Inhibitors
TLR4	Toll-Like Receptor 4
TME	Tumor Microenvironment
TNF-α	Tumor Necrosis Factor Alpha
TRAF2	TNF Receptor-Associated Factor 2
UPR	Unfolded Protein Response
VEGF	Vascular Endothelial Growth Factor
VEGFA	Vascular Endothelial Growth Factor A
VEGFR	Vascular Endothelial Growth Factor Receptor
VEGFR2	Vascular Endothelial Growth Factor Receptor 2
VLDL	Very Low-Density Lipoprotein
Wnt	Wingless-Related Integration Site
XBP1	X-Box Binding Protein 1
XBP1s	Spliced X-Box Binding Protein 1 (active transcription factor)
XIAP	X-Linked Inhibitor of Apoptosis Protein
YAP/TAZ	Yes-Associated Protein/Transcriptional Coactivator with PDZ-Binding Motif
